# Differential exposure to palatable food and its effects on binge-like eating behavior in adolescent rats

**DOI:** 10.3389/fpsyg.2024.1468984

**Published:** 2024-11-21

**Authors:** María Elena Chávez-Hernández, Luis Miguel Rodríguez-Serrano, Daniel Díaz-Urbina, Sinuhé Muñóz-Sánchez, Mario Humberto Buenrostro-Jáuregui, Rodrigo Erick Escartín-Pérez

**Affiliations:** ^1^Psychology Department at the Universidad Iberoamericana Ciudad de México, Ciudad de México, Mexico; ^2^Facultad de Estudios Superiores Iztacala, UNAM. Laboratorio de Neurobiología de la Alimentación, Tlalnepantla, Estado de México, Mexico; ^3^Laboratory on Neurobiology of Compulsive Behaviors, NIMH, National Institutes of Health, Bethesda, MD, United States

**Keywords:** adolescence, palatable food, binge-like intake, hedonic feeding, overconsumption

## Abstract

**Introduction:**

Consumption of palatable food (PF) can lead to chronic overconsumption and obesity. Furthermore, adolescents may be vulnerable to excessively reinforcing foods, which increases the probability of developing overweight and obesity in adulthood. The role of PF availability in binge-like intake among adolescents without caloric needs remains unclear. The present study aimed to evaluate which PF access protocol is the most sensitive to induce increased caloric intake and binge-like eating during adolescence.

**Methods:**

We used 24 male Wistar rats [30 postnatal days (PND)]; standard food and water were provided *ad libitum*. Rats were randomly assigned to one of three groups: (a) continuous, daily access to PF; (b) intermittent, 1-day access/1-day no-access; or (c) weekend, 3 days-access/4 days no-access. All groups had 1 h access to PF (chocolate sandwich cookies). Access protocols were maintained for 6 weeks; afterward, rats underwent a 7-day withdrawal period, and were then evaluated on a binge-eating test.

**Results:**

Chronic restricted PF access induces binge-like intake, with intermittent access resulting in the highest binge index. Additionally, caloric intake of PF increases over time during adolescence, with differential effects of intermittent and weekend access.

**Conclusion:**

Chronic restricted access to PF during adolescence induces binge-like intake, with differences depending on PF availability. This can lead to chronic overconsumption under non-homeostatic conditions.

## 1 Introduction

The control of feeding behavior is regulated by two key systems: homeostatic, necessary for basic metabolic processes and survival, and hedonic, driven by sensory perception or pleasure (Rossi and Stuber, 2018). It is important to note that the hypothalamus, specifically the arcuate nucleus (ARC), is crucial in regulating homeostatic feeding (Ullah et al., 2023). Furthermore, homeostatic regulation control interacts with hedonic controls (Berthoud et al., 2017). In this regard, hedonic hunger, related to hedonic regulation system, occurs in response to a desire to consume food for pleasure; it involves “liking,” which is a hedonic reaction to pleasure, and “wanting,” reflecting incentive motivation (Chmurzynska et al., 2021). Furthermore, hedonic eating, the desire to eat, is driven by the brain’s reward system, and may lead to food addictions and binge eating (de Oliveira et al., 2022). Binge eating is defined clinically as consuming a large amount of food, typically palatable food (PF), during intermittent episodes over a short period of time and a loss of control over what and/or how much is being eaten (Hildebrandt and Ahmari, 2021). Studies have found that hedonic hunger also appears to be closely related to loss of control and binge eating (Espel-Huynh et al., 2018).

In recent years, it has become evident that the hedonic/reward brain system can override the hypothalamic regulation of energy balance, particularly when exposed to a variety of palatable, energy dense, high fat and high sugar foods, leading to food overconsumption beyond homeostatic needs (Amin and Mercer, 2016). In this regard, intake of PF may be a risk factor that can lead to chronic overconsumption, contributing to obesity in non-homeostatic feeding conditions (Sallam and Borgland, 2021; Woodward et al., 2022), and inducing addiction-like deficits in brain reward function, which is considered an important source of motivation that may drive overeating (Johnson and Kenny, 2010). Recent studies have proposed a metabolic-reward circuit, which regulates hedonic intake, and is composed of hypothalamic-mesolimbic pathways, where lateral hypothalamus (LH) signaling activates dopaminergic structures such as the ventral tegmental area (VTA). It is proposed that the Nucleus accumbens (NAc)-LH pathway regulates hedonic intake. The proposed circuit involves the LH, VTA, and NAc, with the latter being a key structure in the regulation of appetitive behaviors (Francke et al., 2019).

Given that adolescence is a particularly sensitive period in central nervous system development, adolescents may be vulnerable to foods that are excessively reinforcing, increasing the risk of experiencing dysregulation of food reinforcement processes from early exposure and over time (Fazzino and Kong, 2022), and may also be more likely to be overweight or obese in adulthood (de Andrade Silva et al., 2021; Kimin et al., 2022). In a study comparing adolescent, adult, and aged rats, adolescent rats consumed more PF in an operant paradigm, both in fixed and progressive ratio, indicating that these rats display a higher motivation to obtain the PF reinforcer (Amancio-Belmont et al., 2017). Additionally, an experiment using adolescent and adult rats showed that limited access to PF three or five times per week resulted in significantly more binge-like eating in adolescents than in adults (Bekker et al., 2014).

It has been proposed that preclinical approaches to the study of binge eating help break it into three clinically-significant parts: (1) consumption of a large amount of food, (2) food consumption over a short period of time, and (3) loss of control over eating (Hildebrandt and Ahmari, 2021). In this regard, access to PF may be a factor that contributes to the first two components of binge-like eating. In this respect, it has been shown that *ad libitum* and continuous restricted access to PF have differential effects on adult male rats, with restricted access inducing binge-like eating behavior to PF, determined as consuming ≥20% of the total daily kilocalories from PF within 1 h access (Muñoz-Escobar et al., 2019). Furthermore, studies have also indicated that young rats exposed to continuous or intermittent access to PF overeat PF on access days (Kreisler et al., 2017; Spierling et al., 2018). Another study showed that exposure to PF during adolescence in short or long operant sessions induces binge-type eating, but the long-access group exhibits significant hyperphagia of PF, indicating that the time of exposure in restricted access models induces changes in PF intake (Curtis et al., 2019). The impact of *ad libitum* and restricted access to PF on binge eating behavior and the risk of overeating under non-homeostatic conditions is well-established. However, it remains unclear how different restricted access protocols to PF can affect these behaviors in adolescent rats. Therefore, the current study aimed to determine which PF access protocol is the most effective in inducing increased PF intake and binge-like eating behavior in adolescent rats.

## 2 Materials and methods

### 2.1 Subjects

Twenty-four thirty-days old male Wistar rats were used for this study. All animals were individually housed to have a precise measure of food intake per animal during the experiment, in a temperature (20°C) and humidity-controlled vivarium, on a standard 12 h light–dark cycle (lights on at 8:00 am and off at 8:00 pm) and had *ad libitum* access to a standard diet (SD; Nutricubos Purina®; 3.36 kcal/g; 23.0% protein, 3.0% fat, and 6.0% fiber) and water. Rats also had 1 h access to PF (chocolate sandwich cookies [Oreo® Nabisco®] 4.67 kcal/g, 4.1% protein, 19.2% fat, and 69.5% carbohydrates) according to the diet protocol. At postnatal day (PND) 25, animals were individually housed and left undisturbed for habituation until starting the experimental protocol on PND 30. Starting on PND 30, animal weight and SD intake were manually recorded every 24 h until the end of the experiment.

All animals used in this study were handled in accordance with the Mexican Official Norm NOM-062-ZOO-1999 technical specifications for the production, care, and use of laboratory animals. All experimental procedures were performed in accordance with the National Institutes of Health Guide for the Care and Use of Laboratory Animals (NIH Publications N°. 8,023, revised in 1978), and local Mexican laws to minimize the number of animals used and their suffering.

### 2.2 Procedure

On PND 30, the rats were randomly assigned to one of three groups: (a) continuous, with daily access to PF; (b) intermittent, with 1 day access/1 day no access; or (c) weekend, with 3 days-access/4 days-no-access. All groups had access to PF for 1 h on access days; PF was presented at 11:00 h. SD and water were removed during PF access, and PF was weighted before and after the 1 h access to register consumption. Outcome measures were body weight, body weight gain, PF caloric intake, and PF binge index.

Body weight was recorded daily, and the body weight gained from the beginning of the experiment was calculated as follows:

$$\mathrm{Body}\;\mathrm{weight}\;\mathrm{gain}=W_{EOW}-W_{\mathrm{start}}$$

where W_EOW_ is the body weight at the end of each week and W_start_ is the weight recorded on day 1 of the experiment (PND30).

Kilocaloric intake was calculated as follows for both PF and SD:

$$\mathrm{Kilocaloric}\;\mathrm{intake}=\left(WF_{\mathrm{placed}}-WF_{\mathrm{found}}\right)\times \mathrm{Kcal}$$

where *WF_placed_* is the weight in grams of the food when first placed in the cage, *WF_found_* represents the weight in grams of the food found in the cage, and *Kcal* is the kilocalories per gram of PF or SD. Additionally, binge-like intake was determined as consuming ≥20% of the total daily kilocalories from PF (Muñoz-Escobar et al., 2019). First, total kilocaloric intake (TOTAL_kcal_) was calculated as the sum of kilocalories from PF and kilocalories from SD:

$$\mathrm{TOTA}L_{\mathrm{kcal}}=PF_{\mathrm{kcal}}+SD_{\mathrm{kcal}}$$

Then, the proportion of PF kilocalories (PF Kcal %) was calculated as follows:

$$PF\;\mathrm{kcal}\%=\left(\frac{\mathrm{PFkcal}}{\mathrm{TOTALkcal}}\right)\times 100$$

The result of this proportion of PF kilocaloric intake was defined and analyzed as PF binge index when the result was 20% or more of daily total kilocaloric intake.

**Figure 1:**
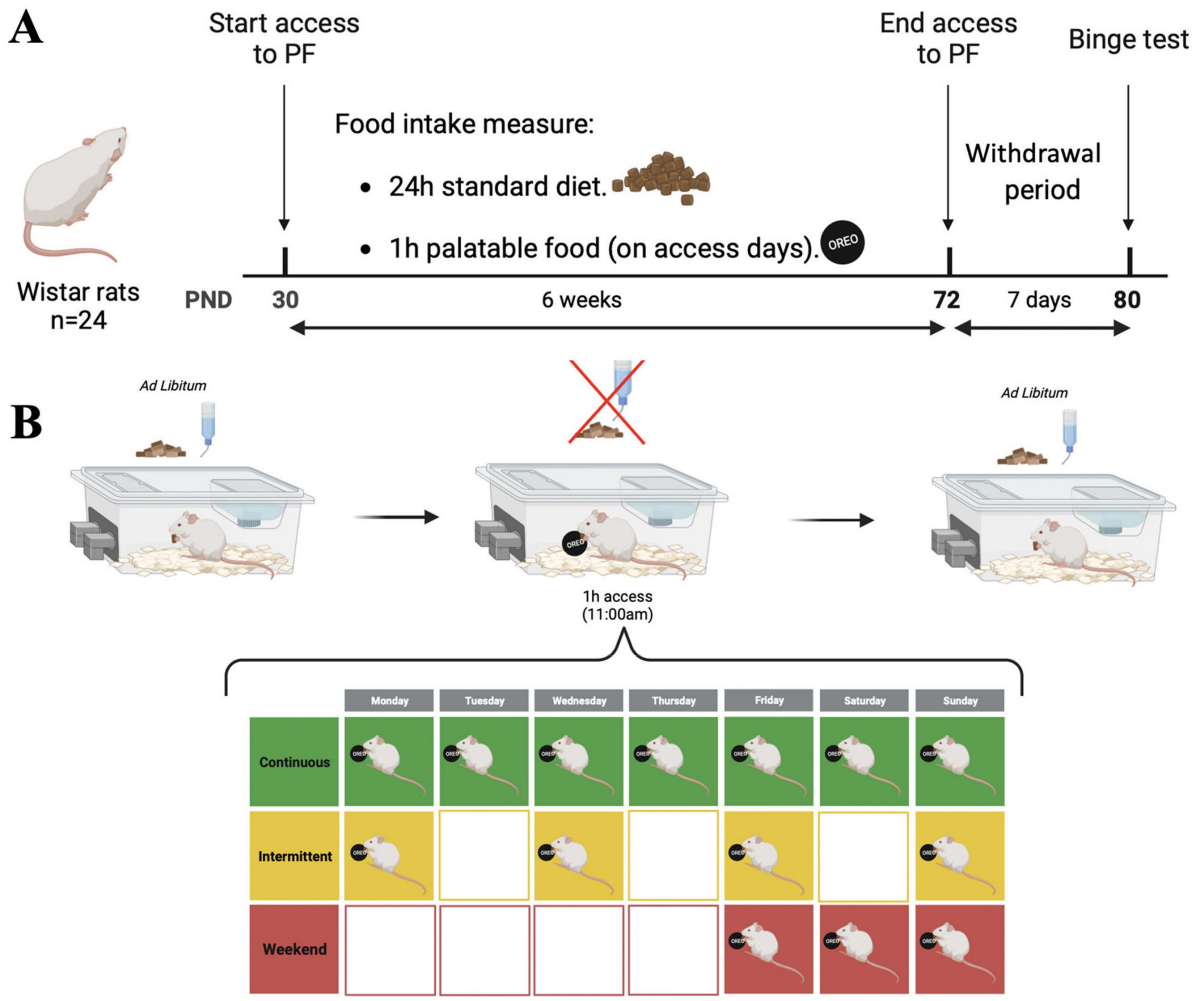
Experimental overview. **(A)** Experimental timeline: Starting on PND 30, rats were assigned to one of three groups: (a) continuous; (b) intermittent, or (c) weekend. All groups had access to PF for 1 h on access days; protocols were maintained for 6 weeks (42 days). After 6 weeks of access to PF, rats underwent a withdrawal period of 7 days and then were evaluated on the binge-eating test. **(B)** Access to PF protocol: Rats had *ad libitum* access to SD and water. During access to PF, SD and water were removed, and after 1 h the remainder of PF was removed, and SD and water returned. All experiments took place in the animal’s home cage. Figure made with Biorender ® software.

The protocols were maintained for 6 weeks (42 days). After 6 weeks of access to PF, the rats underwent a withdrawal (WDL) period of 7 days and were then evaluated on the binge-eating test. Figure 1 shows the experimental overview, including the timeline and access to PF protocols.

### 2.3 Statistical analysis

Data were prepared in Excel and are reported as the mean ± standard error of the mean (SEM). The results were analyzed using GraphPad Prism version 9.3.1 (350) (GraphPad Software LLC, 2021). Figures were generated using GraphPad Prism®.

PF caloric intake and binge-like intake are represented as the mean daily kilocaloric (kcal) intake per day per week. Body weight is represented as mean weight per week, and weight gain is represented as mean grams gained at the end of each week from the starting weight, recorded on PND 30 (experimental day 1).

A two-way ANOVA (group × weeks) with Tukey’s multiple comparisons test (∝ < 0.05) was conducted to compare the main effects of the PF access protocol (group) and time (weeks), as well as their interaction effects on body weight, body weight gain, PF caloric intake, and binge-like intake during the experiment. One-way ANOVA with Tukey’s multiple comparisons test (∝ < 0.05) was conducted to compare binge-eating test results between groups, and changes across time within each group on PF caloric intake, and binge-like intake were analyzed with a one-way ANOVA with Bonferroni’s multiple comparisons test (∝ < 0.05) to compare changes across experimental weeks to week 1.

## 3 Results

### 3.1 Body weight and body weight gain

To analyze the effect that availability of PF across time may have on body weight and body weight gain body weight was recorded daily to obtain a mean weekly weight and compare between groups. A Two-Way (group × time) ANOVA analysis was applied to evaluate the differences between groups on mean weekly body weight and weekly body weight gain.

**Figure 2:**
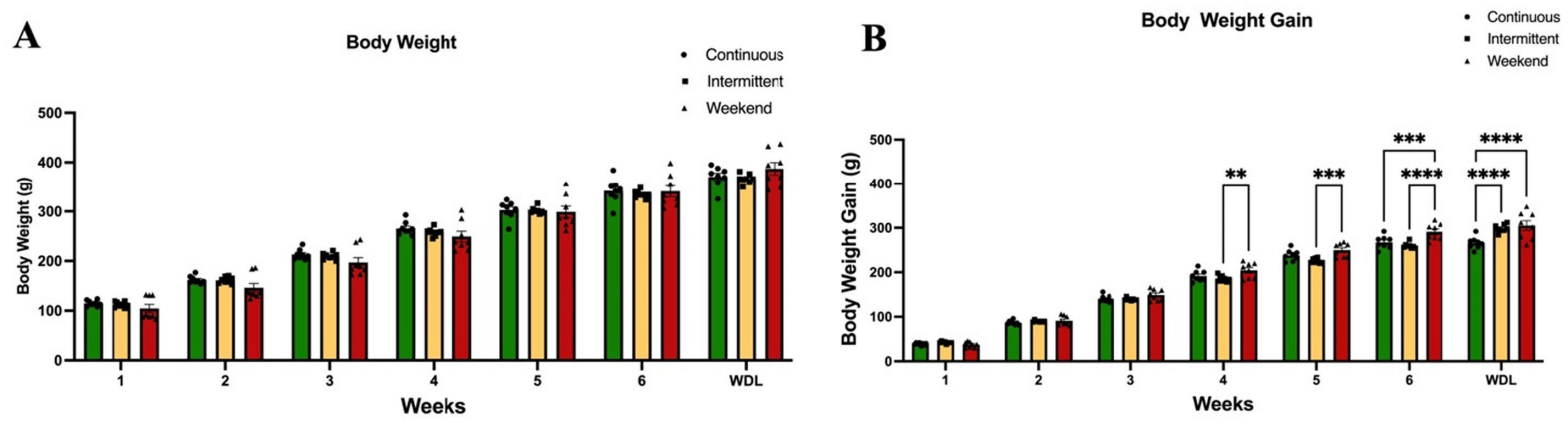
Body weight. **(A)** Body weight per week. **(B)** Body weight gain per week. Data express the mean ± SEM (*n* = 8/group). Two-Way (group × time) ANOVA with Tukey’s multiple comparison tests; asterisks indicate a significant statistical difference between groups (*p* < 0.05). WDL: withdrawal.

The results of the analysis between groups showed that mean weekly body weight remained stable across groups, with no significant weight increases in any of the groups from week to week, for the duration of the experiment (Figure 2A), with no significant interaction effect [*F*_(12, 157)_ = 1.04; *p* = 0.42] among groups, accounting for 0.35% of variance, and no significant effect of access protocol group [*F*_(2, 147)_ = 1.57; *p* = 0.21], explaining 0.09% of variance. A significant effect of time (weeks) was found [*F*_(6, 147)_ = 568.71; *p* < 0.0001], which accounted for 95.45% of the variance; this could be related to normal animal growth given that the experiment started in adolescence (PND 30). However, analysis of body weight gain (Figure 2B) indicated a significant interaction effect [*F*_(12, 147)_ = 5.07; *p* < 0.0001], explaining 0.69% of the variance, and a significant main effect of access protocol group [*F*_(2, 147)_ = 22.21; *p* < 0.0001], accounting for 0.50% of the variance, and time (weeks) [*F*_(6, 147)_ = 1,429.10; *p* < 0.0001], which accounted for 97.14% of the variance.

Tukey’s multiple comparison tests indicated that the weekend group gained significantly more weight than the intermittent group at weeks 4 (*p* = 0.007) and 5 (*p* < 0.0001), on week 6 significantly more than the continuous and intermittent groups (*p* = 0.0005 and *p* < 0.0001 respectively), and on the withdrawal week (both *p* values <0.0001).

### 3.2 Palatable food kilocaloric intake

**Figure 3:**
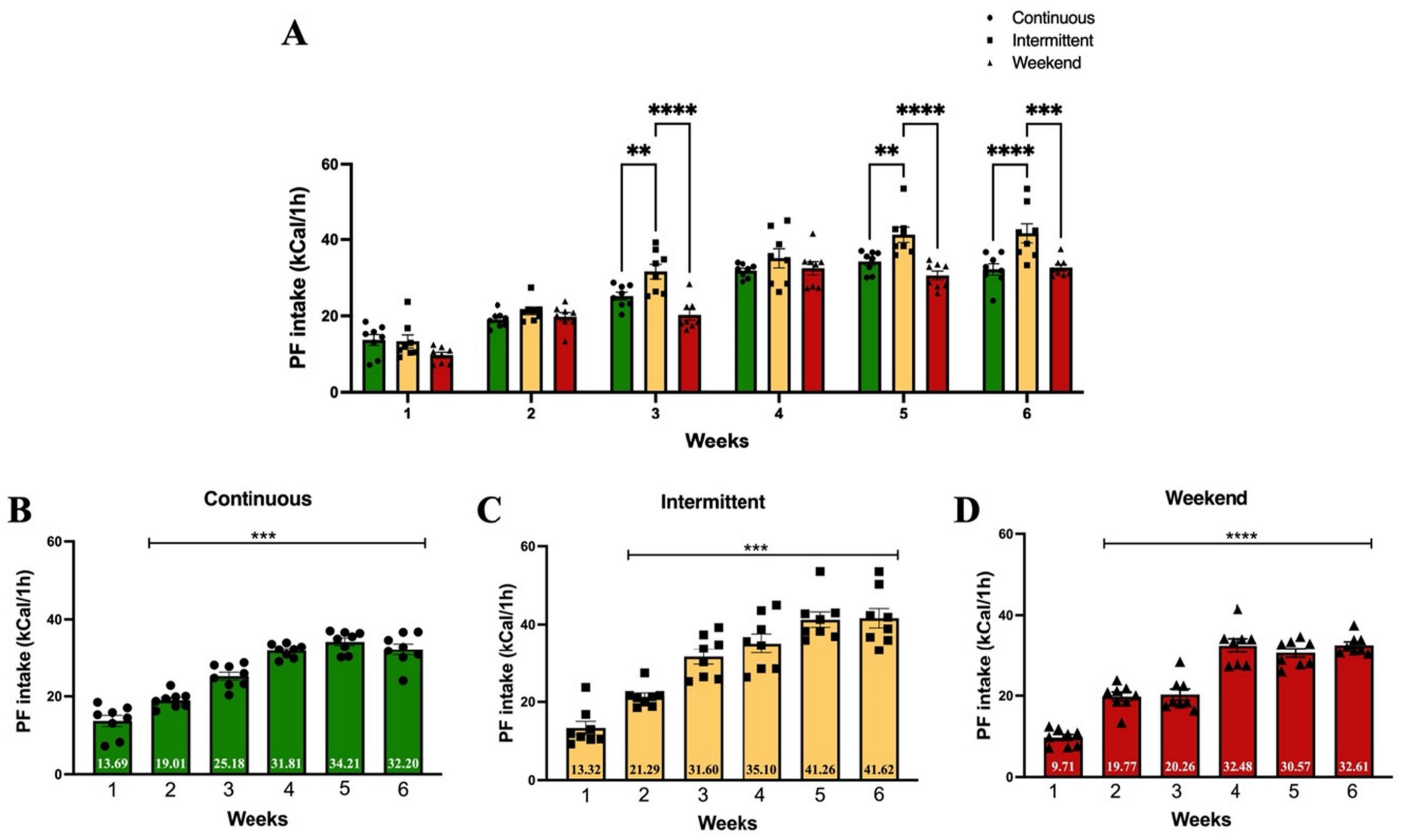
Palatable food kilocaloric intake. Data express the mean ± SEM (*n* = 8/group). **(A)** Two-Way (group × time) ANOVA with Tukey’s multiple comparison tests; asterisks indicate a statistical difference between groups (*p* < 0.05). **(B-D)** One-way ANOVA with Tukey’s multiple comparison test; asterisks indicate a significant statistical difference from week 1 (*p* < 0.05).

Supplementary Figure 1 shows the results of standard diet kilocaloric intake on non-access to PF days for the intermittent and weekend groups. Next, to identify the effect of different access protocols on PF kilocaloric intake, we compared the effect of each access protocol on PF caloric intake using a two-way (group × time) ANOVA. The results showed that all three groups significantly increased their PF kilocaloric intake over the study period (Figure 3A), with a significant main effect of group × time interaction [*F*_(10, 126)_ = 2.93; *p* = 0.0025], explaining 3.57% of variance; there was also a significant main effect of access protocol group [*F*_(2, 126)_ = 30.14; *p* < 0.0001], which explained 7.35% of variance, and time [*F*_(5, 126)_ = 120.90; *p* < 0.0001], explaining 73.71% of variance.

Tukey’s *post hoc* analysis indicated that the intermittent group showed a significant increase on intake in week 3 when compared to the continuous (*p* = 0.008) and weekend (*p* < 0.0001) groups, as well as on weeks 5 (continuous *p* = 0.003; weekend *p* < 0.0001) and 6 (continuous *p* < 0.0001; weekend *p* = 0.0001).

To evaluate changes in PF kilocaloric intake within groups, a one-way ANOVA analysis was conducted comparing PF kilocaloric intake across experimental weeks to caloric intake on week 1 (Figures 3B–D). Results within groups indicated that PF caloric intake significantly increased in the continuous group [*F*_(5, 42)_ = 56.91; *p* < 0.0001] compared to week 1 from week 2 (*p* = 0.007) through week 6 (all *p* values <0.0001); in the intermittent group [*F*_(5, 42)_ = 32.08; *p* < 0.0001] from week 2 (*p* = 0.03) through week 6 (all *p* values <0.0001); and in the weekend group [*F*_(5, 42)_ = 58.03; *p* < 0.0001] from week 2 through week 6 (all *p* values <0.0001).

### 3.3 Binge-like eating of palatable food

**Figure 4:**
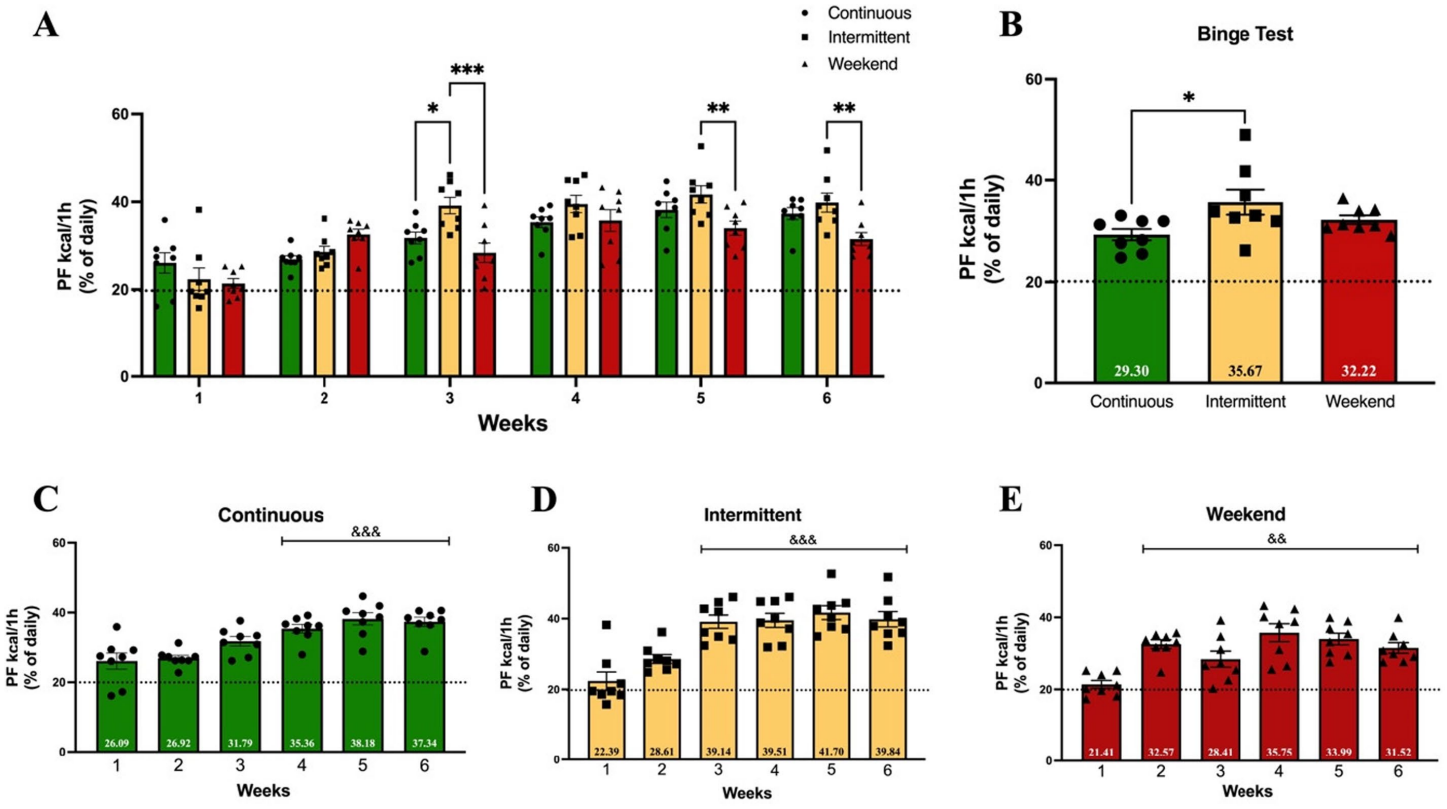
Binge-like intake (≥20% of daily kcal consumed from PF during 1 h access). Mean PF binge-like intake during 1 h on access days and after 1 week of withdrawal (Binge Test). Bars indicate the proportion of the total mean daily caloric consumption from PF. Data express the mean ± SEM (*n* = 8/group). **(A)** Two-way (group x time) ANOVA with Tukey’s multiple comparison test; asterisks indicate a statistical significant difference between groups (*p* < 0.05). **(B)** One-way ANOVA with Tukey’s multiple comparison test; asterisks indicate a statistical significant difference between groups (*p* < 0.05); **(C-E)** One-way ANOVA with Tukey’s multiple comparison test; asterisks indicate a statistical significant difference from week 1 (*p* < 0.05).

Previous studies indicate that access to PF plays an important role in overeating and binge-like eating, with differential effects of continuous or intermittent access to PF (Kreisler et al., 2017; Muñoz-Escobar et al., 2019; Spierling et al., 2018). In the present study, we compared the effects of three access protocols (continuous, intermittent and weekend) to evaluate which protocol is the most sensitive in inducing binge-like intake during adolescence. A two-way (group × time) ANOVA analysis was conducted on the binge index of PF, which was established as consuming ≥20% of the total caloric intake of PF in 1 h on access days. Results showed that all three groups surpassed the binge index starting on week 1 (Figure 4A). Results of two-way (group × time) ANOVA analysis of binge-like intake indicate that the interaction effect was significant [*F*_(10, 126)_ = 3.24; *p* = 0.0009], explaining 9.89% of the total variance, and also a significant main effect of access protocol group [*F*_(2, 126)_ = 10.06; *p* < 0.0001], explaining 6.14% of the variance, and time (weeks) [*F*_(5, 126)_ = 29.87; *p* < 0.0001], which explained 45.55% of the variance. Tukey *post hoc* test results indicated that on week 3, the intermittent group showed a significantly higher binge-like intake than the continuous (*p* = 0.01) and weekend (*p* = 0.0001) groups on week 3, and significantly higher than the weekend group on weeks 5 and 6 (*p* = 0.007 and *p* = 0.003 respectively).

To evaluate changes across time in binge index within groups, a one-way ANOVA analysis was conducted (Figures 4C–E). Binge-like intake significantly increased in the continuous group [*F*_(5, 42)_ = 11.59; *p* < 0.0001] when compared to week 1 on week 4 (*p* = 0.0005), week 5, and week 6 (both *p* values <0.0001); in the intermittent group [*F*_(5, 42)_ = 15.21; *p* < 0.0001] on weeks 3 through 6 (all *p* values <0.0001); and in the weekend group [*F*_(5, 42)_ = 8.51; *p* < 0.0001] from week 2 through week 6 (all *p* values <0.0001).

After a 7-day withdrawal period, the rats underwent a Binge Test (Figure 4B) to evaluate persistence of binge-like intake after a non-access to PF period. A One-way ANOVA was conducted to evaluate differences between groups. Binge Test results showed that there were differences among groups [*F*_(2, 21)_ = 3.82; *p* = 0.04], with the intermittent group presenting the highest binge index with a significant difference when compared to the continuous group (*p* = 0.03).

## 4 Discussion

The present study aimed to evaluate which PF access protocol is the most sensitive to induce increased intake and binge-like eating behavior of PF during adolescence. Our results show that all access protocols induce binge-like intake, and that the intermittent access model is the most sensible to provoke this type of eating behavior, as evidenced by a significantly different weekly escalation in the binge index, as well as significantly more intake during the binge test. These results are consistent with previous studies that indicate that intermittent 3 times/week access for 1 h to PF significantly increases intake when compared to a daily restricted access model in adult rats (Vazquez-Herrera et al., 2021). Furthermore, it has also been reported that short (30-min) intermittent 3 times/week access to a PF significantly increases binge-like eating behavior when compared to continuous access (Kreisler et al., 2017) or continuous 5 times/week access (Bekker et al., 2014), and that after 4 weeks of 3 times/week feeding protocol, food intake during the access period increases to approximately 70% of chow-only 24-h energy intake (Corwin and Wojnicki, 2006), even though rats are not food deprived. In addition to these findings, our study also included a weekend access model (three continuous times/week), which interestingly showed lower PF caloric and binge-like intake than the continuous (non-significant) and intermittent access models. It is important to note that the results of the continuous group (1 h of access daily) are also in line with previous studies that demonstrated that binge-like eating is induced in restricted daily access models to PF (Muñoz-Escobar et al., 2019; Zepeda-Ruiz et al., 2020). Additionally, intermittent presentations of PF have been shown to increase binge-like eating, in adult rats when compared to continuous access (Wojnicki et al., 2008). Nevertheless, these studies started the access to PF in adult rats. In this regard, our results provide a novel approach showing that during adolescence and adulthood (PND 30 through 72) the development of binge-like eating is related to PF access.

On the other hand, our results show that increased binge-like intake depends on the type of exposure of PF, not necessarily on caloric needs, and that access protocol to PF can lead to caloric overconsumption (Berland et al., 2022). This indicates that the development of binge-like intake in adolescents rats is not dependent on caloric restriction, which is the same as in real-life situations, and is *per se* a risk factor in the development of obesity in adults (Bekker et al., 2014). Usually, caloric restriction is used to improve motivation for binge-like eating behavior (Placidi et al., 2004; Zepeda-Ruiz et al., 2020), a condition where homeostatic control of food intake is essential for survival; additionally, PF may produce powerful changes in the brain reward circuitry that we did not evolve for, leading to overconsumption (Morin et al., 2017). Our study shows that binge-like intake increases in a chronic exposure from adolescence to adulthood according to PF availability without an apparent caloric need, given that rats are not food deprived. The findings from our study also show in a preclinic model that PF overconsumption in adolescents rats among adolescent rats is not solely driven by caloric necessity, indicating that PF has a high reward salience that can lead to consumption and overconsumption, given that it can occur in sufficient energy reserve conditions (Harb and Almeida, 2014). This result may provide insight into the neurobiological mechanisms that underlie overconsumption.

Studies on binge-like intake during adolescence have mainly focused on alcohol intake, and few studies have evaluated the binge-like intake of PF during this critical period. In this regard, it is important to note that adolescence demands higher nutritional and requirements, and poor diet quality and eating habits established during this period can have long-term consequences, including obesity (Mumena et al., 2023; Wahl, 1999), and eating patterns and behaviors during this period are influenced by many factors, including food availability (Das et al., 2017). The effects of early chronic exposure to PF in restricted access protocols during this period are of particular interest in understanding the development of binge-like eating over time. In this regard, it has been demonstrated that binge-like sucrose intake in adolescent rat models reduces dendritic length and complexity of principal neurons in the basolateral amygdala (Novelle and Diéguez, 2018). Our preclinical study provides useful information on how chronic restricted access to PF in these three models increases binge-like eating in adolescent rats.

It has also been shown that caloric restriction can induce binge-like eating behavior in mice in a 2 h period (Hambly and Speakman, 2015). Regarding PF, the time of exposure to PF has the strongest effect on increasing caloric intake, weight gain, and binge-like intake, and long-term intermittency has been shown to promote larger binge-like intake of PF (Kreisler et al., 2017). Also, restricted access to PF has shown to rapidly induce binge-like intake, with briefer access periods leading to greater intake on initial time of access (Curtis et al., 2019; Kreisler et al., 2018). In our study, results confirm that access plays a salient role in binge-like eating.

Our study also identified the effects of early consumption of PF in adolescent rats and indicated that the longer the exposure to intermittent and daily access to PF, the stronger the effects on these measures, and that it also induces overeating. This is helpful in understanding the effects of long-term consumption of PF during adolescence, suggesting that overconsumption strongly depends on PF access, even without caloric needs, and that these effects continue to grow over time.

It is also important to note that in preclinical studies, limited access models of animal binge-like eating provide a useful tool to evaluate behavioral changes with regard to restricted high sucrose/fat/mixed diets, whereas chow and water are free access (Rospond et al., 2015). In this regard, it has been shown that restricted access to PF can increase its consumption and induce binge-like eating in adult rats (Boggiano et al., 2007; Espitia-Bautista and Escobar, 2021), but little is known about the effects of chronic access to PF during adolescence.

Our preclinical limited access study provides helpful information on the distinct effects that three restricted access models to PF have on binge-like eating behavior when rats are chronically exposed during adolescence. Additionally, our results indicate that food access protocol appears particularly important for increasing energy intake, and for the development and intensity of binge-like eating behaviors, which contributes to understanding how chronic access to PF in different access protocols alters these measures when starting in early adolescence.

Regarding binge-like intake after a withdrawal period, it has been shown that rats exposed to a 2-day access/5-days no access to PF for 7 weeks showed compulsive eating when renewing access to PF, indicating that this behavior alleviates a withdrawal-induced negative emotional state (Iemolo et al., 2012). Our results indicated that rats with intermittent access showed the highest binge index in the Binge Test after a 7-day withdrawal period, which may indicate the highest withdrawal symptoms in this group after 6-weeks of alternating 1-day access/1 day no-access to PF, compared to the weekend group that underwent 4-days with no access for the 6 weeks protocols were maintained. Interestingly, the continuous access group showed the lowest binge-index during the test, confirming that chronic intermittent access to PF induces negative emotional effects, and renewing access relieves withdrawal-induced negative affects (Iemolo et al., 2012), indicating that rewarding and hedonic effects of PF may result in a positive emotional reaction, reducing negative states, playing a major role in overeating and obesity (Singh, 2014).

PF qualities are also important for inducing increased intake and binge-like eating. In this regard, Tenk and Felfeli (2017) analyzed the intermittent access of PF for a single ingredient or a combination (sweetness and fat), and showed that combined PF consumption is significantly higher than that of a single ingredient (Tenk and Felfeli, 2017). Furthermore, a study by Kendig et al. (2022) showed that a solid diet produces more marked effects on energy intake than liquid sugar; in their study, rats were chronically exposed to a solid cafeteria diet (high-fat high-sugar) and/or a 10% sucrose water solution, finding that all rats exposed to cafeteria diet had significantly increased energy intake when compared to rats exposed only to a 10% sucrose water solution and control rats (Kendig et al., 2022). In our study, the PF provided (chocolate sandwich cookies) combines high-sugar, high-fat and solid diet qualities, which offers a strong model of PF to evaluate energy intake and binge-like eating behavior.

Finally, the findings from the present study suggest that different access protocols to PF impacts differentially in the body weight gain, and this effect depends on the developmental stage. Moreover, our data indicates that adolescent rats are undergoing a normal growth development, which is important to consider. Future studies may be conducted to analyze how PF kcal intake is correlated to body weight and body weight gain during this development. Furthermore, the present study included only male subjects, a potential limitation. However, research indicates that the prevalence of eating disorders in men has increased in the last few years (Gorrell and Murray, 2019; Halbeisen et al., 2022), and that some developmental variables of the BED do not differ between men and women (Barry et al., 2002). In this regard, we consider that our results support valuable evidence about the effect of different diet PF access protocols on BED development during adolescence. Sex differences in these results may be explored in future studies. Our findings also highlight that after withdrawal, higher binge like eating index persisted only in rats intermittently exposed to the PF, suggesting that mechanism controlling food seeking and craving may be driven the binge relapse. Further studies are needed to fully understand the neurobiological mechanism controlling hedonic responses to the orosensory properties of the PF, and how that increases the risk for development of overweight and obesity.

In summary, PF intake is mainly related to hedonic responses related to the orosensory properties of the foods (e.g., caloric content, taste). In the present study, even when sated, rats consumed PF in a binge-like manner, suggesting that rats consumed the PF by its intrinsic value instead of homeostatic needs. Different access paradigms to PF can be helpful in studying binge-like intake, which is related to food addiction and binge eating disorder. In addition, determining the most sensible access protocol during adolescence can be helpful in studying how other variables (e.g., stress) may alter PF intake in hedonic feeding regulation from early life stages. Furthermore, understanding the impact of other factors, such as food availability, physical activity, and genetic factors on PF intake during adolescence is crucial in elucidating the complex interplay between developmental, cognitive, and emotional factors that shape feeding behavior.

## Data Availability

The raw data supporting the conclusions of this article will be made available by the authors, without undue reservation.
